# Artificial intelligence inferred microstructural properties from voltage–capacity curves

**DOI:** 10.1038/s41598-022-16942-5

**Published:** 2022-08-04

**Authors:** Yixuan Sun, Surya Mitra Ayalasomayajula, Abhas Deva, Guang Lin, R. Edwin García

**Affiliations:** 1 School of Mechanical Engineering, Purdue University, West Lafayette, USA; 2 School of Materials Engineering, Purdue University, West Lafayette, USA; 3 Department of Mathematics, Purdue University, West Lafayette, USA

**Keywords:** Batteries, Computational methods

## Abstract

The quantification of microstructural properties to optimize battery design and performance, to maintain product quality, or to track the degradation of LIBs remains expensive and slow when performed through currently used characterization approaches. In this paper, a convolution neural network-based deep learning approach (CNN) is reported to infer electrode microstructural properties from the inexpensive, easy to measure cell voltage versus capacity data. The developed framework combines two CNN models to balance the bias and variance of the overall predictions. As an example application, the method was demonstrated against porous electrode theory-generated voltage versus capacity plots. For the graphite|LiMn$_2$O$_4$ chemistry, each voltage curve was parameterized as a function of the cathode microstructure tortuosity and area density, delivering CNN predictions of Bruggeman’s exponent and shape factor with 0.97 $R^2$ score within 2 s each, enabling to distinguish between different types of particle morphologies, anisotropies, and particle alignments. The developed neural network model can readily accelerate the processing-properties-performance and degradation characteristics of the existing and emerging LIB chemistries.

## Introduction

Lithium-ion batteries, LIBs, are a well-established energy storage technology, powering a wide range of small scale applications, including smartphones and laptops, as well as large scale applications, such as electric vehicles and grid storage. The LIB market is continuously growing due to the increase in global demand for renewable energy storage and elimination of greenhouse gases^1,2^. In the last two decades, LIB technology has greatly evolved^3^, creating new chemistries and architectures that have been optimized for many applications in cost effective ways.

A key design factor to develop reliable, optimal LIBs is the fabrication of new electrode microstructures^4–9^. Specifically, microstructural features such as active material particle size, shape, alignment and distribution have a direct impact on the battery performance^10,11^. These microstructural features are traditionally quantified as a function of volume fraction left by the solid electrode material phase referred to as the porosity, $\varepsilon$, which in turn controls the reactive area density, ${\mathcal {A}}$, defined as:

1 $$\begin{aligned} {\mathcal {A}}={S}(1-\varepsilon )/r_p \end{aligned}$$

where $r_p=(3V_p/4\pi )^{1/3}$, is the size of a characteristic particle of volume, $V_p$. *S* is the shape factor of the electrode particles and a function of the electrode particle morphology, distribution, and alignment^7,11^.

Another key microstructural parameter associated to battery performance is the tortuosity, $\tau$, which is related to the electrical conductivity and chemical diffusivity of porous electrodes through the expression $D= D_\circ \varepsilon /\tau$, where $\tau$ is given by the Bruggeman relation^7,12,13^:

2 $$\begin{aligned} \tau = 1/{\varepsilon }^{\alpha } \end{aligned}$$

Here, $\alpha$, is the Bruggeman exponent and captures the coarse-grained contributions of the particle size distribution, particle morphology, distribution, and alignment^11,12,14–16^.

In spite of the importance of the microstructural properties of LIBs and their impact on the associated performance, degradation, and cost, to the best of the authors’ knowledge, the quantitative estimation of battery properties remains expensive, slow, and difficult to measure. Experimentally, battery microstructural parameters are currently inferred from reconstructing tomographic images^5,7,10,14,15,17–25^. For example, Shearing et al.^17^ highlighted microstructural heterogeneities through X-ray tomographic imaging, by considering different volume sizes of an electrode sample and found that larger volumes are more representative of the entire sample. Ebner et al.^5^ determined the particle size distribution in LiNi$_{1/3}$Mn$_{1/3}$Co$_{1/3}$O$_2$, NMC, electrodes and quantified porosity due to composition of additives and compaction pressure during manufacturing and found that the discharge capacity at high C-rates was unaffected by the compaction pressure but increased with additives. Chung et al.^10^ calculated tortuosity using tomographic experimental data^5^, and computer generated electrodes to show the particle size distribution and packing affect on the tortuosity and area density. In that study, experimental electrodes displayed a 15% higher tortuosity than computer generated electrodes, demonstrating the importance of establishing processing-microstructural properties correlations of LIB electrodes.

Ebner et al.^7^ used NMC, graphite, and LiCoO$_2$, LCO, electrodes with different particle shapes to demonstrate for the first time the critical effect that morphological anisotropy has on tortuosity. A factor of three increase was found for through-thickness versus in-plane tortuosity for the electrodes. Müller et al.^24^ analyzed porosity, particle size distribution, tortuosity, and area density of four commercial graphite electrodes and found that the local ratio of porosity and tortuosity induced localized voltage drops in the electrode potential during charging. Pietsch et al.^25^ estimated the uncertainty in determining microstructural parameters and found variabilities as high as 200% for low porosity electrodes, demonstrating their importance in defining a high quality electrode layer.

Although tomography experiments are effective in determining the microstructural parameters, they require an immense economic and computational effort to process and prepare the electrode layers, image the resultant samples, and post process the resultant images^14,19,21,26^. In particular, tortuosity estimation requires additional lengthy calculations on the reconstructed images^10,14,22,27,28^. Other experimental techniques to infer tortuosity include AC impedance-based methods, the polarization interrupt method, e.g., see Thorat et al.^29^, where the tortuosity is determined from the effective chemical diffusivity, and the blocking electrolyte method, e.g., see Landesfeind et al.^30^, where the tortuosity is determined from the effective electrical conductivity. Pouraghajan et al.^26^ compared these methods and proposed a generalized impedance based model. These methods require experimental processing of the electrode sample under investigation and some of them require further fitting of electrochemical models to data^31^. Overall, existing non-destructive microstructural quality battery characterization approaches seem impractical to be run jointly and on-the-fly with the production line.

In contrast, machine learning, ML, techniques have been applied to infer multiple aspects of battery technology^32^, including the state of health, SOH, where the aim is to estimate the remaining useful life of the battery, RUL, and optimize battery operating conditions^33,34^. ML models for batteries include regression based methods^35,36^, support vector machines, SVMs^37^, Markov chain and Monte Carlo methods^33,34,38–42^. Recently, neural network based approaches have been gaining importance to monitor SOH and RUL in real time^34,42^. Zhang et al.^43^ used long short-term memory recurrent neural networks, RNN, to predict the RUL by learning the long term dependencies in degradation data. Typically, RNN and their variants have been used to predict SOH and RUL^43–48^, wherein they are trained against cell voltage, current, and temperature^34,34,35,37,43,44^. In addition, charge capacity has been estimated through regression models^49,50^. Specifically, the state of charge, SOC, has been estimated by using deep learning methods and establishing correlations between voltage, current, temperature, power, and energy of the LIB during voltage discharge^51–58^.

ML techniques have also been employed in battery materials discovery^59–62^. Techniques such as regression and neural networks are used to predict material properties such as electrical conductivity and reaction rates^63–65^. Sendek et al.^60,66^, implemented a regression model to screen potential lithium ion conducting solid state electrolyte materials, while Jalem et al.^67^, implemented a neural network. Ahmad et al.^68^, trained a graph convolutional neural network, CNN, to screen for electrolyte materials that suppress lithium dendrite growth. Joshi et al.^69^, used a neural network, SVM, and regression analysis to predict the open circuit voltage of electrode materials. Jiang et al.^70^, developed a ML model to determine the statistics of NMC electrode particle and binder detachment based on the X-ray tomography data. Badmos et al.^71^, implemented deep learning and CNN to detect defects in LIBs for quality assessment. In all these cases, data generation and availability are among some of the major concerns for ML modeling in LIB technology^32,72^.

Currently, the emergence of ML-based tools has focused on estimating the electrochemical state, the degree of degradation, or the prediction of new materials. In contrast, in this paper, a convolutional-dense hybrid neural network-based model has been developed to infer from experimental voltage versus capacity data, the microstructural properties that determine battery performance characteristics within a few seconds, instead of hours or days, by using what would currently be considered expensive, destructive, and slow characterization techniques. This sets the stage to accelerate the processing-properties-performance and degradation characteristics, of the existing and emerging chemistries.

## Methodology

**Figure 1:**
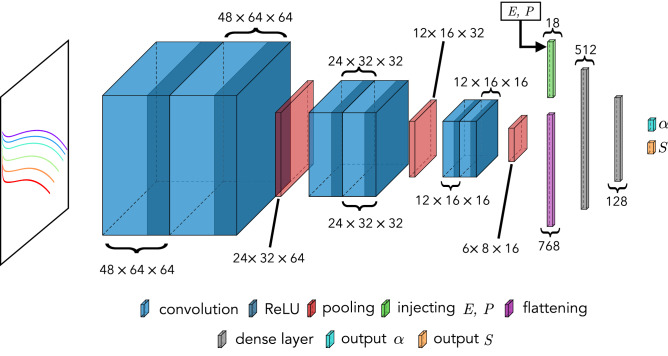
Convolutional neural network architecture to infer microstructural battery parameters. The CNN is comprised of convolution blocks and fully connected layers, which takes two types of input at different stages. The model takes the color-encoded voltage versus capacity curves as the main input (each color corresponding to a current density), energy density, *E*, and power density, *P*, as the second input. Each convolution block has two convolutional layers, followed by a pooling layer. A ReLU activation function is placed after each convolutional layer and hidden dense layer. For each data point, the image with voltage curves are fed into the network. For each curve, *E* and *P* are taken into the following fully connected layers, along with the higher-level representation of the input image. The output of this network has two components, the Bruggeman exponent, $\alpha$, and the area density shape factor, *S*. See text for details.

Figure 1 shows the proposed neural network combined with a convolutional and dense layers approach. Here, an image containing color-coded normalized voltage curves, each color corresponding to a different current density, and the associated energy density, *E*, and power density, *P*, are used as inputs. At the input layer, the convolutional kernels extract higher level representations. A down-sampling max-pooling layer follows each convolutional block, reducing the input size for the next layer, achieving translation invariance and preserving the important information, see Table 1.

**Table 1:** Network architecture description.

Operation layers		Number of filters	Kernel size	Stride	Padding	Output size
Input voltage curves	–	–	–	–	–	$48 \times 64 \times 3$
Convolution layer	ReLU	64	$3 \times 3$	$1\times 1$	Same	$48\times 64\times 64$
Convolution layer	ReLU	64	$3 \times 3$	$1\times 1$	Same	$48\times 64\times 64$
Pooling	max pooling	–	$2\times 2$	$2\times 2$	Same	$24\times 32\times 64$
Convolution layer	ReLU	32	$3 \times 3$	$1\times 1$	Same	$24\times 32\times 32$
Convolution layer	ReLU	32	$3 \times 3$	$1\times 1$	Same	$24\times 32\times 32$
Pooling	max pooling	–	$2\times 2$	$2\times 2$	Same	$12\times 16\times 32$
Convolution layer	ReLU	16	$3 \times 3$	$1\times 1$	Same	$12\times 16\times 16$
Convolution layer	ReLU	16	$3 \times 3$	$1\times 1$	Same	$12\times 16\times 16$
Pooling	max pooling	–	$2\times 2$	$2\times 2$	Same	$6\times 8\times 16$
Extra input (E, P) and flattening	ReLU	–	$3 \times 3$	$2\times 2$	Same	$18+768$
Dense layer	ReLU	–	–	–	–	512
Dense layer	ReLU	–	–	–	–	128
Output $\alpha$	–	–	–	–	–	1
Output *S*	–	–	–	–	–	1

Because the aim is to predict $\alpha$ and *S* with the same neural network, two challenges are noted: (1) the need to minimize the bias towards *S* during training, where target ranges are drastically different (﻿$\alpha$: 0–5; *S*: 0–40); and (2) the tendency of neural network to favor higher *S*-values for mean squared error ﻿($L_2$ loss). Therefore, two identical CNN models with customized loss functions were combined.

Two CNN models were developed, the first one is referred herein as the $L_{\mathrm {w}}$-model, and uses a weighed mean squared error, $L_{\mathrm {w}}$, which is the loss function:

3 $$\begin{aligned} L_{\mathrm {w}} = \sum _{i=1}^{n} \frac{W_{\alpha }(y_i - \hat{y}_i)^2_{\alpha } + W_{S}(y_i - \hat{y}_i)^2_{S}}{n} \end{aligned}$$

$y_i$ is the true value and $\hat{y}_i$ is the model prediction for the *ith* voltage versus capacity curve and *n* is the total number of data points. $W_{\alpha }$ and $W_S$ control the importance of each quantity during model prediction.

The second network corresponds to the same architecture trained with another loss function, defined herein as the $L_{\mathrm {M}}$-model, to reach a uniform relative error for the entire target span and to emphasize the lower $\alpha$ and *S* values, where:

4 $$\begin{aligned} L_{\mathrm {M}} = \frac{1}{n}\sum _{i=1}^{n} \frac{| y_i - \hat{y}_i|}{|y_i|} \end{aligned}$$

To evaluate the performance of the models, the coefficient of determination, $R^2$, was used to account for the proportion of the true target variance:

5 $$\begin{aligned} R^2 = 1 - \frac{\mathrm {V}_{res}}{\mathrm {V}_{tot}} \end{aligned}$$

where $\mathrm {V}_{res} = \sum _{i=1}^{n} (f_i - y_i)^2$ and $\mathrm {V}_{tot} = \sum _{i=1}^{n} (y_i - \bar{y})^2$. Here, $f_i$ represents the predicted values from trained neural network, $y_i$, represents the true values in the pre-processed dataset, and $\bar{y}$ is the sample mean of $y_i$.

$L_{\mathrm {s}}$ measures the averaged relative error and is defined as:

6 $$\begin{aligned} L_{\mathrm {s}} = \sum _{i=1}^{n} \frac{|y_i - \hat{y}_i|}{0.5(y_i + \hat{y}_i)}\times 100\% \end{aligned}$$

to represent the percentage deviation between the predicted and the true values. The residuals were normalized against their mean and standard deviation to fairly compare the model performance to predict $\alpha$ and *S*. A combination of these two models was implemented to balance the bias and variance.

## Numerical implementation

Voltage vs. capacity curves were generated using dualfoil.py, an open source python software, developed by Robinson and García^73^, which python-wraps the dualfoil legacy fortran code made publically available by Doyle et al.^74^. Different combinations of the Bruggeman exponents and shape factors of the cathode were sampled while the values corresponding to other design adjustable and material parameters were kept constant. The Bruggeman exponent was discretized into intervals of 0.1, ranging from 0 to 10, which correspond to experimentally observed ranges^10,11^. The shape factor was discretized into intervals of 1, ranging from 0 to 40, also in agreement with experimentally observed particle morphologies^6,10,16^. The discharge currents were varied from 1.75 A m$^{-2}$ to 122.5 A m$^{-2}$, for each combination of shape factor and Bruggeman exponent, resulting in a total of 15,600 simulations. 1500 simulations that did not converge were discarded without affecting the resolution of the dataset, see Deva and coworkers for details^16^. A 2.0 V cutoff voltage was set, and the energy and power density values were extracted for each voltage curve as additional input parameters.

Dualfoil.py can currently model without any modifications, electrode materials such as $\hbox {LiMn}_2$O$_4$, $\hbox {LiCoO}_2$, graphite, and TiO$_2$ but it could include new electrode chemistries, if needed^73^. For the $\hbox {LiMn}_2$O$_4$ and graphite chemistry pair, the dualfoil code simulates experimental current densities as high as 105 A m$^{-2}$^74^. The dualfoil code employs a homogenization approximation for upscaling microscopic equations^74,75^ thus, its veracity would be limited at higher current densities for all cell chemistries^76^. During data generation, dualfoil.py simulation time was $\sim$20$s-$300 s per current density curve for a given combination of Bruggeman exponent and shape factor.

The proposed neural network architecture was implemented in Tensorflow, trained on a Tesla P100 GPU on 12 hours of wall time. Tenfold cross validation was used to report the model performance where, in each fold, the entire dataset was split randomly into ten equal parts: the network was trained on nine parts and evaluated on the remaining part. In order to assess the validity of the predicted $\alpha$ and *S*, these values were used as input into dualfoil.py , to predict the associated voltage versus capacity curves. The final compound neural network combined both the $L_{\mathrm {w}}$-model and $L_{\mathrm {M}}$-model by reporting the predicted values of greater performance for each model, given their range of validity.

## Results and discussion

As an example application, a $\hbox {LiMn}_2$O$_4$, and graphite electrode cell was used to train the CNN. In general, the analysis and data curation process requires the sampling of a statistically representative section of the microstructural parameter space and the corresponding voltage *vs* capacity response, given an imposed set of current densities. This process can be readily performed by carefully fabricating porous electrode microstructures of tailored tortuosity and area density^7,16,17,20^, and by filling the microstructural parameter gaps by using well developed physics-based microstructural models. In the case of LIBs based on porous electrode layers, the microstructural response has been well described in terms of the well established porous electrode theory model, as pioneered by Newman et al.^77^, Doyle et al.^74,75^, and developed by a well-established community, e.g.,^78–81^. Specifically, through the use of dualfoil.py, the space of microstructural parameters can be readily explored, e.g., see Deva et al.^16^.

**Figure 2:**
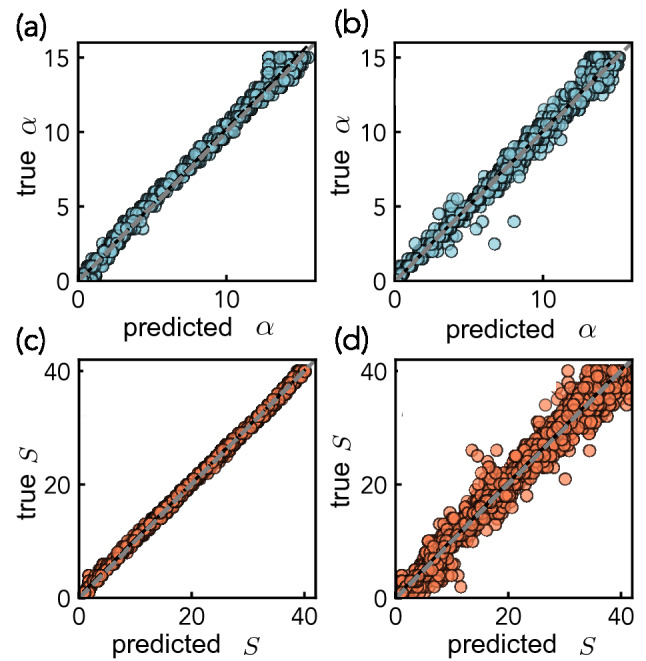
Aggregated true and predicted scatter plots from tenfold cross validation for (**a**) Bruggeman’s exponent $\alpha$ from $L_{\mathrm {w}}$-model with $L_{\mathrm {s}} = 5.59 \%$ and $R^2 = 0.99$, (**b**) $\alpha$ from $L_{\mathrm {M}}$-model with $L_{\mathrm {s}} = 2.95 \%$ and $R^2 = 0.99$, (**c**) shape factor *S* from $L_{\mathrm {w}}$-model with $L_{\mathrm {s}} = 0.89 \%$ and $R^2 = 1.0$, and (**d**) *S* from $L_{\mathrm {M}}$-model with $L_{\mathrm {s}} = 2.59 \%$ and $R^2 = 0.97$. Overall, the trained model accurately predicts both $\alpha$ and *S*. Specifically, the $L_{\mathrm {w}}$-model performed better at predicting *S* with 1.70% less error throughout the range of *S* values, while the $L_{\mathrm {M}}$-model was better at predicting $\alpha <3.0$ by over 5% in comparison to the $L_{\mathrm {w}}$-model. A combination of the model predictions was adopted for the final prediction.

Figure 2 directly compares the expected or true $\alpha$ and *S* values against the neural network predictions, showing a 99% model explained variance. The $L_{\mathrm {M}}$-model delivers a 2.95% error for $\alpha$ and $R^2 > 0.99$. The $L_{\mathrm {w}}$-model resulted in higher $L_{\mathrm {s}}$ values because the $L_{\mathrm {w}}$-model is sensitive to large errors from high *S* and $\alpha$ values. Figure 2a, b shows that for large $\alpha$ values, the points lie more close around the identity line, while for low $\alpha$ and *S* values, the points deviate from the line. Similarly, Fig. 2c, d show that there is at least 97% of the variance the trained model can explain in the true distribution.

**Figure 3:**
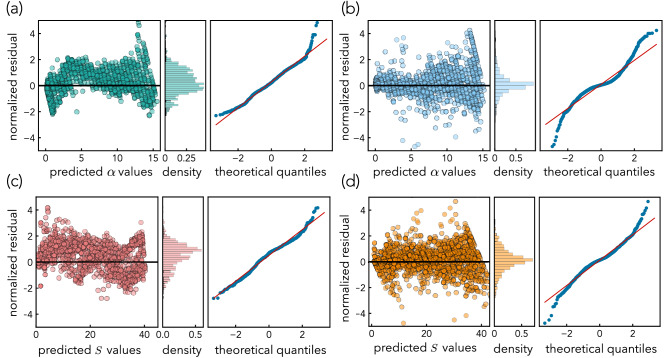
Residual analysis of the proposed models, showing the normalized residual plots, their densities, and the corresponding Q–Q plots. (**a**) $\alpha$ residuals from the $L_{\mathrm {w}}$-model as given by Eq. (3). Results show that $L_{\mathrm {w}}$-model underpredicts by $\sim$ 3% for $3<\alpha <12$ and overpredicts by 5.0% otherwise. The mean of the residuals is greater than zero, i.e., overall the model underpredicted $\alpha$. The corresponding Q–Q plot suggests a near symmetric Gaussian distribution of residuals with a slight right-skew. (**b**) $\alpha$ residuals from the $L_{\mathrm {M}}$-model shows the residuals are more centered around zero with larger values than the $L_{\mathrm {w}}$-model. The corresponding Q–Q plot indicates a near symmetric Gaussian distribution of residuals with heavy tails. (**c**) *S* residuals from the $L_{\mathrm {w}}$-model shows an overall underprediction. The associated Q–Q plot shows Gaussian distribution of residuals with a slight right-skew. (**d**) Shows the residuals of *S* from the $L_{\mathrm {M}}$-model are centered around zero. The corresponding Q–Q plot suggests a near symmetric Gaussian distribution of residuals with heavy tails.

In Fig. 3, each inset shows the normalized residual, its density, and the corresponding normal quantile–quantile (Q–Q) plot for $\alpha$ and *S* of the aggregated values of the tenfold cross-validation for both $L_{\mathrm {w}}$- and $L_{\mathrm {M}}$-models. The models deliver residuals with a Gaussian distribution supporting an unbiased ML model. The Q–Q plots for the $L_{\mathrm {w}}$-model suggest the residuals display near symmetric Gaussian distributions with a slight skew to the right for predicting $\alpha$ and *S*. In contrast, the mean of residuals from the $L_{\mathrm {M}}$-model for both $\alpha$ and *S* was closer to zero and their Q–Q plots indicate heavy tails compared to the Gaussian distributions with the same mean and variance.

**Figure 4:**
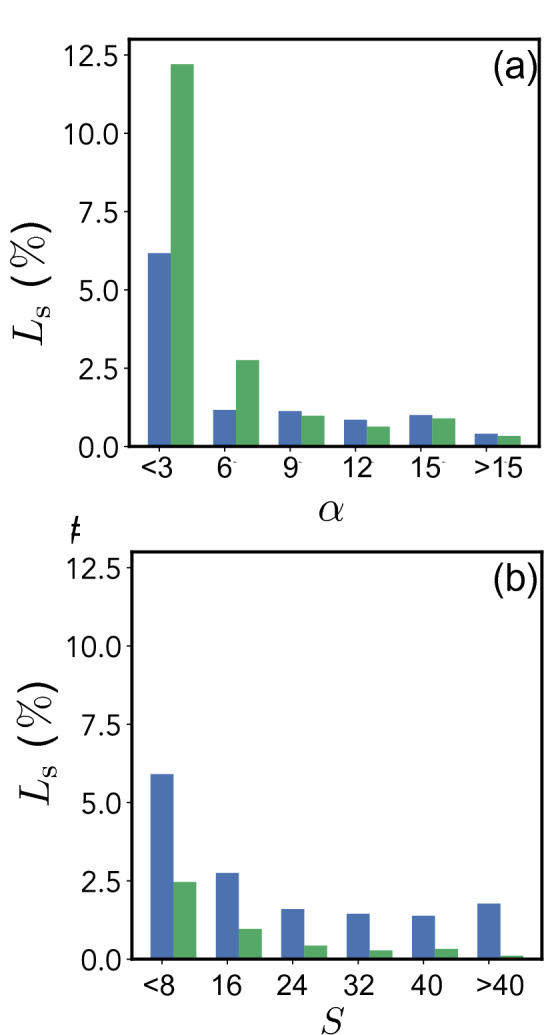
$L_{\mathrm {s}}$ as computed from the tenfold cross-validation from the $L_{\mathrm {w}}$-model (green) and $L_{\mathrm {M}}$-model (blue) performance. (**a**) Shows the effect of $\alpha$. (**b**) Shows the effect of *S*. A lower $L_{\mathrm {s}}$ value means the model prediction is better in that range of values.

Figure 4 shows the performance of both $L_{\mathrm {w}}$- and $L_{\mathrm {M}}$-models for different output ranges. Figure 4a shows that the $L_{\mathrm {M}}$-model delivers a better prediction for $\alpha <6$, the physically realistic range of values for porous electrode LIBs. For $\alpha >6$, both models deliver a similar performance with the $L_{\mathrm {w}}$-model returning a lower $L_{\mathrm {s}}$ value.

**Figure 5:**
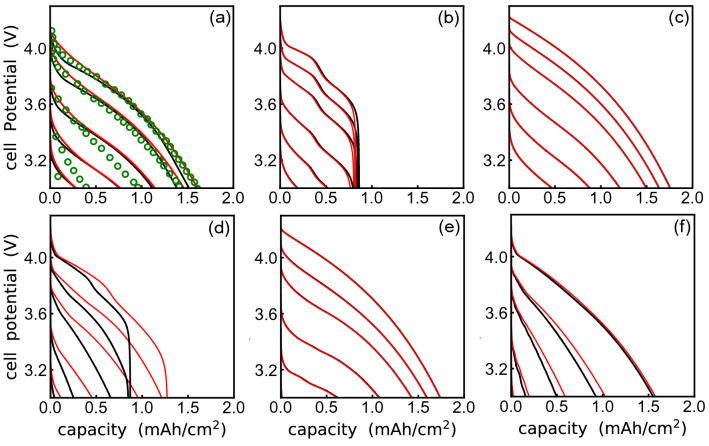
Expected, , and CNN-predicted, , galvanostatic behavior for representative battery microstructures. Inset (**a**) compares experimental data, , and traditional porous electrode theory response, as reported by Doyle and Newman^74^, by using traditionally assumed values, $\alpha =1/2$ and $S = 3$, while CNN-predicted values are $\alpha =2.37$ and $S = 11.12$. In inset (**b**) expected values corresponds to $\alpha =0.05$ and $S = 1.0$, while CNN-model generated values are $\alpha =0.12$ and $S = 0.96$. The root mean squared, RMS, deviation in galvanostatic behavior of the CNN-model prediction with respect to the expected values, show a value of 1.5%. Inset (**c**) corresponds to dual porous structure with low porosity, with expected values of, $\alpha =0.05$ and $S = 40$, while the CNN-model generated values are $\alpha =0.05$ and $S = 39.05$. The RMS deviations are less than 0.05 %. Inset (**d**) corresponds to a distribution of highly textured (aligned, MRD > 20), morphologically anisotropic particles (c/a $\sim$ 1/10) with expected values of $\alpha =6$ and $S = 1.0$, while the CNN-model generated parameters are $\alpha =5.70$ and $S = 1.48$. A maximum RMS deviation of 13.3% is observed. Inset (**e**) expected values are, $\alpha =6$ and $S = 40$, and the CNN-model predicted values are, $\alpha =6.03$ and $S = 39.22$. The RMS deviations are less than 0.15%. Inset (**f**) expected values correspond to $\alpha =8$ and $S = 5$, while the CNN-model predicted values are $\alpha =7.65$ and $S = 4.37$. The maximum RMS deviation is 5.5 % and the minimum RMS deviation is 0.67%.

In contrast, Fig. 4b shows that the $L_{\mathrm {w}}$-model delivered a lower $L_{\mathrm {s}}$ value across the entirety of the trained ranges, and thus was chosen as the model to make predictions for *S*. Both $L_{\mathrm {w}}$- and $L_{\mathrm {M}}$-models perform well on predicting $\alpha$ (see Eq. 2) and *S* (see Eq. 1), but the $L_{\mathrm {w}}$-model under-predicts, suggesting it has a higher bias compared to the $L_{\mathrm {M}}$-model. However, the $L_{\mathrm {M}}$-model delivered larger residuals, indicating higher variance. Therefore, a combined model that returns the best output from each $L_{\mathrm {w}}$- or $L_{\mathrm {M}}$- model, as they sample different bins and different accuracies will enable a better prediction and a larger winning margin.

Figure 5 compares the cell potential versus capacity electrochemical behavior that results from different battery microstructures against the combined model. Specifically, inset (a) compares the experimental behavior, as reported by Doyle and Newman^74^, against their dualfoil prediction using the traditional spherical limit approximation, i.e., $\alpha = 1/2$ and $S= 3$, and the values predicted by the CNN by using the experimental voltage response as input. Not only the CNN-generated microstructural parameters provide a better match to the experimental response ($\sim$2.2% error of the spherical limit versus $\sim$0.8% for the CNN-base prediction at low current densities), but the graphically-inferred values, $\alpha = 2.37$ and $S=11.12$, demonstrate that the shape of the particles are morphologically anisotropic and display a great degree of surface area, as one would expect in a real microstructure. Further, while the CNN prediction is highly accurate, particularly for low current densities, the simulation demonstrates that at high current densities, polarization losses dominate the response of the cell, regardless of the particle morphology. The deviations between model and experiment are a result of model limitations unable to capture the particle-particle effects that result at high current densities, e.g., see Battiato et al.^76^.

For dual porous battery architectures, e.g.,^82^, Fig. 5b, c highlight the effect of area density on the predicted electrochemical response, showing that the CNN can easily distinguish between high and low quality designs. In particular, inset (b) shows that the low power density design is result of a subpar area density delivered by the dual porous microstructure. In contrast, inset (c) shows that a dual porous, bicontinuous architecture cannot deliver very high power densities, but can out perform the traditional layered porous design.

For traditional single porosity, electrode designs conformed of highly aligned (textured) platelets (MRD > 20), with morphological anisotropy, c/a $\sim$ 1/10, but poor area density, inset (d) shows that even though the CNN captures the relevant features controlling the microstructural electrochemical behavior, there are some instances where differences between the expected and predicted ($\alpha , S$) pairs can lead to a 25% difference in the overall predicted charge capacity. In this specific case, the difference is a result of a 48% difference in the *S*-value. However, for the same microstructural properties, an increase in area density of 20$\times$ delivers a match by the CNN-model that is virtually indistinguishable from the expected behavior, see inset (e). Further, insets (d) and (e) demonstrate the possibility of inferring from voltage measurements in porous electrodes the same particle morphology-induced tortuosity but a widely different electrochemically active area density, enabling the possibility of tracking the area density losses that result from degradation, such as those resulting from decrepitation^83^ or SEI growth^84^. Further, the effects of different powder qualities, e.g., different particle morphological anisotropies and their processing-induced alignment^7^, and the corresponding area densities, can be easily inferred through the proposed CNN-model, enabling the possibility to distinguish even subtle differences, compare insets (e) and (f), whose quantification is critical for the advanced fabrication of energy storage technology.

## Conclusion

A convolution neural network-based deep learning model was presented to infer porous electrode microstructure properties from the macroscopic voltage behavior, specifically, Bruggeman’s exponent, $\alpha$, and shape factor, *S*, by starting from six voltage versus charge capacity response curves, each for a different current density as well as the corresponding power and energy density. Two models were trained using adjusted $L_{\mathrm {w}}$ and $L_{\mathrm {M}}$ loss functions, and were combined to produce a combined model that accurately predicts microstructure properties.

The developed CNN-framework allows to distinguish between different types of particle morphologies, anisotropies, and particle alignments, as well as the effects on the area density. All of these microstructural characteristics are a result of processing, including powder selection, layer compaction, and calendaring, and are key to specify the quality of the processing operation. As presented, the developed methodology can be readily incorporated into the battery production process as a step to track the microstructural quality of the developed product and assert control on the developed energy storage technology. Further, the developed CNN-model can be readily used as a way to estimate the amount of active material left as a result of the multiple cycle-induced microstructural changes on the voltage versus charge capacity response as a result of the electrochemically active area density loss and increase of the electrode impedance.

Finally, while we used computer-generated data to demonstrate the ability of the CNN model to predict battery microstructural parameters from voltage versus charge capacity curves, the methodology can be easily implemented by using a statistically representative, carefully fabricated set of battery architectures that span a physically realistic range of processing parameters, and can be readily extended to infer other relevant battery design parameters, such as layer thickness, particle size, lithium diffusivities, electrical conductivities, etc. The availability of curated, public databases that have carefully labeled the microstructural parameters, as well as voltage and capacity response will be key to apply this formulation to the generality of battery chemistries and designs. The Jupyter notebook associated to the model can be accessed on Google Colab. The source code can be accessed at microbattAI.
